# Hospital-Associated Transmission of *Brucella melitensis* outside the Laboratory^1^

**DOI:** 10.3201/eid2101.141247

**Published:** 2015-01

**Authors:** Christopher F. Lowe, Adrienne J. Showler, Suzette Perera, Susan McIntyre, Roohi Qureshi, Samir N. Patel, Vanessa Allen, H. Roslyn Devlin, Matthew P. Muller

**Affiliations:** University of Toronto, Toronto, Ontario, Canada (C.F. Lowe, A.J. Showler, R. Qureshi, S.N. Patel, V. Allen, H.R. Devlin, M.P. Muller);; St. Michael’s Hospital, Toronto (S. Perera, S. McIntyre, R. Qureshi, H.R. Devlin, M.P. Muller);; Li Ka Shing Knowledge Institute, Toronto (H.R. Devlin, M.P. Muller);; Public Health Ontario, Toronto (S.N. Patel, V. Allen)

**Keywords:** Brucella, Brucella melitensis, nosocomial infection, health care workers, laboratory-acquired infection, transmission, bacteria

## Abstract

*Brucella melitensis* was identified in an aspirate obtained from a patient’s hip joint during a procedure at a hospital in Canada. We conducted an investigation into possible exposures among hospital workers; 1 worker who assisted with the procedure tested positive for *B. melitensis*. Aerosol-generating procedures performed outside the laboratory may facilitate transmission of this bacterium.

Brucellosis is the most common laboratory-acquired infection (*1*,*2*), and laboratory acquisition has been estimated to account for up to 2% of all *Brucella* infections (*3*). Infection rates among laboratory workers after exposure to *Brucella* spp. have been reported to be as high as 30% (*4*,*5*), although recent investigations have described lower attack rates (0–3.8%) (*6*–*9*). This difference may be the result of a broader definition of exposure, improved laboratory safety standards, and prompt administration of antimicrobial prophylaxis. Even so, laboratory personnel have experienced severe brucellosis manifestations such as osteomyelitis, meningitis, and death (*9*). Therefore, manipulation of *Brucella* isolates should occur under Biosafety Level 3 conditions. However, this practice is challenging to implement in developing countries because of lack of resources and high incidence of infection and in industrialized countries because of a low clinical suspicion for brucellosis. In response to the ongoing occurrence of laboratory exposures, the Centers for Disease Control and Prevention (CDC) issued guidelines for the identification and management of laboratory workers potentially exposed to *Brucella* spp., including recommendations for prophylaxis (*9*,*10*).

## The Study

In July 2012, aspiration of the hip joint of a patient with suspected prosthetic hip infection was performed in the interventional radiology department (normal pressure, 9 air exchanges/hour) at St. Michael’s Hospital in Toronto, Ontario, Canada. Personnel wore gloves but not masks or facial protection. Straw-colored synovial fluid was aspirated into a sterile container. At the time of the procedure, *Brucella* infection was not suspected, although retrospective review showed that the patient had risk factors, including regular travel to India (most recent trip 2 months before the aspirate sample was taken) and consumption of unpasteurized buffalo milk in India. Because the patient was lost to follow-up, we were unable to obtain informed consent for a detailed case description.

The synovial fluid culture was sent to the microbiology laboratory; documentation did not indicate that *Brucella* spp. was a possible etiologic agent. All microbiology specimens at the laboratory are initially processed under a class II biological safety cabinet. Initial Gram stain testing of the sample showed polymorphonuclear cells but no bacteria. On day 3, scant growth of small white colonies was observed on sheep’s blood agar and chocolate agar but not on MacConkey agar; Gram stain testing showed gram-negative coccobacilli. The organism was positive for oxidase and catalase, but testing with VITEK2 (bioMérieux, Marcy l’Etoile, France) did not identify the organism. By that time, the sample had been on an open bench for 6 days, and the sample was then referred to the provincial reference laboratory. There, the organism was identified as *B. melitensis*—18 days after the specimen was initially obtained and 10 days after it was sent to the reference laboratory.

After *B. melitensis* was identified, we initiated an investigation to identify laboratory personnel who may have been exposed to or infected with the bacterium. CDC recommendations from 2008 were used to classify laboratory personnel into high- and low-risk categories and to guide prophylaxis and follow-up (*9*). Although the CDC guidance did not address the management of exposure among nonlaboratory health care workers (HCWs), we considered HCWs who were in the procedure room during the aspiration to be at high risk. A total of 12 persons were identified as high risk (10 from the laboratory, 2 from radiology); 20 laboratory personnel were identified as low risk.

All HCWs classified as high risk completed prophylaxis with 3 weeks of doxycycline (100 mg orally 2×/d) and rifampin (600 mg orally 2×/d). Serial serologic testing at baseline, 2, 4, 6, and 24 weeks after *B. melitensis* was identified was recommended for all 32 exposed HCWs. Serologic testing for IgG was performed at the provincial reference laboratory by using an in-house serum tube agglutination test. Thirty HCWs (10 high-risk and 20 low-risk) completed the serial serologic testing (Table); however, the baseline serologic tests were obtained ≈3 weeks after the initial exposure because of the delay in identifying *Brucella* in the culture of the aspirate. 

**Table:** Baseline serologic test results for 32 health care workers exposed to *Brucella melitensis*, Toronto, Ontario, Canada, 2012

Risk and test result categories	Test results, no. workers (titer, if applicable*)
High-risk, radiology, n = 2	
Negative†	1
Indeterminate	0
Positive	1 (1:160)
High-risk, laboratory, n = 10	
Negative	9
Indeterminate	1 (1:80)
Positive	0
Low-risk, laboratory, n = 20	
Negative	18
Indeterminate	1 (1:80)
Positive	0
Refused testing	1

*Negative, ≤1:20; interdeterminate, 1:40–1:80; positive ≥1:160. †Lost to follow-up, but serologic results were negative at 6 weeks after exposure.

A radiology technician classified as high-risk (held the specimen container during the procedure and assisted with injecting the synovial fluid from a syringe into a container) had serologic test results positive for *Brucella*. Her initial titer, obtained 3 weeks after exposure, was 1:160. She began antimicrobial drug prophylaxis 2 days after the initial sample was sent to the laboratory; on the basis of the positive result, drug therapy as noted above was extended to 6 weeks. A repeat titer 8 weeks after exposure was 1:320, and further titers performed at 24 weeks–20 months after exposure remained elevated (>1:160). Two years postexposure, she remains asymptomatic. She consented to have her epidemiologic and clinical data used in this report.

All HCWs with negative baseline results remained negative for the duration of follow-up. Two staff members who had indeterminate titers had no known risk factors for *Brucella* infection other than the laboratory exposure and remained asymptomatic for 24 weeks with stable titers.

## Conclusions

*Brucella* is a well-recognized cause of occupationally acquired infection among microbiology laboratory staff. However, *Brucella* infection was not suspected in this case because of its rarity both in Canada and as an etiologic agent in prosthetic joint infections (*11*). The delay in organism identification and completion of aerosolizing procedures (e.g., catalase test) on an open laboratory bench increased the risk for exposure among hospital workers. Prompt identification and prophylactic treatment of high-risk laboratory staff members prevented clinical disease and seroconversion.

Previous descriptions of *Brucella* outbreaks have focused on laboratory-associated exposures (*2*,*6*,*7*,*9*,*10*). Synovial fluid is considered a low-risk specimen for *Brucella* exposure because the number of organisms is low and the risk for transmission is reported to be minimal compared with exposure to purified organisms in the laboratory (*1*). In this instance, however, transmission was theoretically possible: aspiration of the joint and forceful ejection of the synovial fluid from a syringe into a sterile container could result in aerosolization. 

The HCW with positive serologic test results was a radiology technician who assisted in the procedure. She was born in Egypt and immigrated to Canada in 2003, but she had no subsequent travel back to Egypt or other *Brucella*-endemic areas and no other risk factors for infection. Her elevated titers might have occurred because of past exposure in a *Brucella*-endemic country, but she had left Egypt 9 years prior, and her titers would be expected to be low, even if she had a distant history of *Brucella* infection. Furthermore, in *Brucella*-endemic countries, serum agglutination titers >1:160 are considered positive (*12*). Her titers were elevated on subsequent testing to as high as 1:320, but the serologic response may have been blunted by prompt antimicrobial drug treatment.

Hospital-associated transmission of *Brucella* outside of the laboratory setting may represent a rare occurrence. Two cases of transmission from mother/child to obstetrician have been described (*13*,*14*). Similar criteria for investigating exposures were used in a case of a *B. abortus* hip infection in the operating room but did not identify any transmissions (*15*). However, cases identified in *Brucella*-endemic areas may have been attributed to community, rather than occupational, exposure.

On the basis of our findings, we recommend that HCWs performing aspiration or other aerosolizing procedures on patients with known or suspected *Brucella* infection should use fit-tested N95 respirators and other appropriate personal protective equipment, including gloves, gown, and facial protection. If exposure occurs without the use of appropriate protective equipment, monitoring and serologic follow-up should be initiated, as well as possible prophylaxis for those at highest risk (e.g., performing procedure, holding specimen). Follow-up is critical in non–*Brucella*-endemic areas because the incubation period is prolonged, clinical suspicion may be low, and the potential for delayed diagnosis in the event of illness is high.
